# The Central N. Y. Homœopathic Medical Society

**Published:** 1883-03

**Authors:** C. P. Jennings

**Affiliations:** Secretary


### THE CENTRAL NEW YORK HOMŒOPATHIC MEDICAL SOCIETY.

A quarterly meeting of the Central New York Homœopathic Medical Society was held in Syracuse, N. Y., December 21st, A. D. 1882, seventeen members being present.

Julius Schmitt, M. D., of Rochester; Park Lewis, M. D., of Buffalo, and H. D. Baldwin, M. D., of Syracuse, were admitted to membership, being recommended by the Committee on Credentials.

On motion, Erie County was included in the territory of this Society.

The Secretary reported that, according to instructions of the last meeting, he selected a drug in the 30th trituration for proving; that it was furnished gratuitously by a trustworthy pharmacist, and that thirty phials of the drug were distributed to members of this Society. A summary of the provings will be made when the reports are all gathered in.

Dr. Clausen: Dr. A. Lippe, in a recent letter to me, suggests an inquiry as to whether this Society might think it advisable to protest against the resolution of the American Institute, adopted at its last meeting, on freedom of medical opinion.

On motion, the suggestion of Dr. A. Lippe was accepted. The Chair appointed Drs. Clausen, Hawley, and Hussey a committee to prepare a protest and report it to the next meeting.

Natrum muriaticum was made a subject of conversation, members to state what they had accomplished with it.

Dr. L. B. Wells: A lady, æt. 65, had a tertian fever; came at 10 A. M.; paroxysms increased in severity. She had never had intermittent fever before this attack. Natrum muriat., CM (Swan's), lessened the severity of the paroxysms and finally extinguished the fever.

Dr. Brewster: Have used Natrum mur. successfully in intermittent fevers. A man, æt. 60, had a tertian ague; chill at 9 or 10 A. M., lasting an hour; then fever three hours; intense headache; a sort of paralysis of limbs, so that he would lie as if dead; thirst during the chill; the fever subsided with a moderate sweat; had suffered four or five paroxysms. Natrum mur.[30] cured him in about four days. Suspended the medicine during the paroxysm.

A middle-aged lady complained of having a bad cold; lips and nasal alæ thickly set with fever blisters. Natrum mur. cured promptly.

Dr. C. L. Swift: A lady sick with intermittent fever; chill at 10 A. M.; throbbing headache. Natrum mur.[6] in water, a dose every two hours, cured.

Dr. Boyce: Natrum mur. and Lachesis are the pillars of Homœopathy. In Long Island City there are streets built up, but many of the lots are deep pits full of water. A lad had been there as a visitor. On returning from there to his home he sickened. He became sallow, anæmic; suffered from boils and running sores. Friends became seriously uneasy. Boy not likely to live. His physicians resorted to tonics. He grew worse. A council was called. I recommended Natrum mur.[300]. Three doses were given. In three days the worst was over. He came right up. My experience with Natrum mur. has been in cases abused by Quinine and Arsenic, and my experience has been very happy. Notwithstanding our daily use of it in food, the high potencies of it are effective.

Dr. Hussey: Have cured fever and ague with Natrum mur. more frequently than with any other drug. Repeatedly have I succeeded with it in one or two days. Have found the indications for its use to be the same which others have mentioned here. Have used the 8 M successfully in cases of nasal catarrh, accompanied with excessive use of salt, even when the salt was not discontinued.

Dr. Clausen: Have had but few opportunities of testing it. Recall one case of tertian ague. Chill at 10 A. M.; excessive thirst in the chill; headache. Natrum mur.[200], three pellets of size No. 40, cured. A few days after the patient took the remedy a crop of pearls came out on the lips—on the lower lip chiefly. The patient had never had the sores before.

Dr. Baldwin: Have cured several cases of intermittent fever with Natrum mur. The patients had taken Quinine. Fever blisters were present on the lips; paroxysms came on at 10 or 11 A. M.

Dr. Hawley: Have cured with Natrum mur. a marked desire for salt. March 5th, 1880, a gentleman came to me for treatment. He had suffered from malarial fever in South America, on the Amazon River. He had taken Quinine in quantities. He found it necessary to take it, else every three weeks an indescribable pain would seize the knees, followed the next day by a chill. The discomfort in the knees began in the afternoon, and the chill the next day began in the afternoon also. He had taken Quinine for years. Gave him one dose of Natrum mur. CM (Fincke), which had been medicated in 1868. A week later gave him a second dose of the same. It had become a case of quinine cachexia. The cure was speedy. His paroxysms had two striking characteristics of the medicine—the pain in the head and relief from sweat.

Had a case a few weeks ago of excessively painful urination. The patient had a morbid appetite for salt. Natrum mur.$^{400}$ cured the urinary trouble and lessened the appetite for salt. I do not think salt especially responsible for catarrhs.

Dr. Marks: Have used Natrum mur. frequently, and always with success when indications were clear. Chill comes at 10 or 11; thirst in all stages; a very severe headache; all symptoms relieved by sweat. Have not used higher than the 200th. Hydroa on the upper lip is a characteristic symptom.

Dr. Seward: Have used it often with success when indicated by the symptoms which have been named here.

Dr. Wallace: Have done many good things with it when symptoms pointed to it, such symptoms as have been described here.

Dr. Hussey: A lady was treated allopathically three years for fever and ague. She bought Quinine by the ounce and thought she must take it all the time. She had become cachectic. Friends prevailed upon her to consult me. Natrum mur. 8M, twelve doses, a dose every night for twelve nights, cured.

Dr. Wells: Have found Natrum mur. important in delaying catamenia in young girls. Have had fine success with it.

Dr. Brewster: A young lady with the monthly flow upon her desired to go to a ball. She suppressed the flow by putting her feet in cold water. The consequences declared themselves. She lost her health. Two or three years afterward she became my patient. General debility, headache, backache, scanty menses, in countenance pale, in form slender. Gave her occasionally a dose of Natrum mur. 2M (Fincke). She recovered and is now a large, robust woman. The cure required several months, but it remains permanently.

Dr. Jennings: A boy of 16 had a tertian ague. First paroxysm comparatively light. The headache and his Belladonna constitution led to giving him a dose of Belladonna.[30] The next paroxysm was severe. It came on about 10 or 11 A. M. Two hours after the subsidence of the fever Natrum mur.[30] one pellet, was exhibited. No return of chill and fever. Recovery was quick and complete—and this occurred in the Wabash Valley. Have seen chronic diseases of fever and ague, which have been treated many months with Quinine, Arsenic, and patent medicines, yield readily to one dose of Natrum muriat.[200] (Dunham). Dose not to be repeated unless there should be another paroxysm, and that paroxysm as severe and long-continued as the preceding one. Repetition called for very seldom.

Dr. Seward read a clinical paper on a case cured with Lachesis.

Dr. Wallace: A case of rheumatism in the left side, going from one joint to another, crossing over to the right side. High fever and restlessness suggested Aconite. Lachesis 5M cured.

Dr. Boyce: Migration from left to right is a key-note of Lachesis.

Dr. E. P. Hussey reported on Neural Analysis. His report included a paper prepared by Dr. Fincke, of Brooklyn, L. I., also Professor Jæger's analysis. The thanks of this Society were given to Dr. Fincke for his paper. Dr. Hussey was requested to prepare a digest of the paper of Dr. Fincke's and of Profe sor Jæger's analyses, and report to the Society. The following statement is a summary of Dr. Hussey's report:

Neural analysis is a method of detecting and measuring very slight deviations in the nerve-force of a person. There are two methods: one discovered by Professor Jæger, of Stuttgart, Germany, in which Hipp's chronoscope, an electric clock designed to measure exceedingly minute divisions of time (previously used for astronomical purposes), is ingeniously made to measure the time occupied by a sensation in passing from one part of the body to another; the other method, discovered by Dr. Fincke, of Brooklyn, is by means of a very delicately constructed galvanometer, with which, the human body being the battery, the slightest variation of the nerve-force is exactly measured. Experiments by different observers give some very interesting results, among which are regularly varying conditions of the nerve-force at certain hours of the day, it being depressed in the morning after rising, increased somewhat after breakfast, followed by a depression again before noon, increased again after dinner, and decreased in the evening to still lower figures. There is a decrease after a nap, and also, invariably, from cold temperature and from fatigue. Mental and muscular activity, any special excitement, music, etc., produced decided rise. Every kind of food and drink, and even each different odor, produces its particular effect. Drugs have each its own effect. The most interesting fact is the effect upon the nerve-force produced by taking the finer, and even the finest, homœopathic preparations of drugs, those which have hitherto been considered by most physicians as belonging to the realm of the imaginary or the mythical, high potencies being proved by these instruments capable of producing more marked effects than the lower potencies. These results will stimulate investigation to discover the unexplained law of expansion, which makes it possible for highly attenuated medicines to affect the human organism so powerfully.

Dr. Hawley: If we can get, in this way, decided proofs of the effect of potencies on the well and on the sick it will be of great benefit to Homœopathy. It would be well for the younger members of the Society to prepare instruments and experiment and study the subject.

Dr. Boyce: Dr. Fincke's instrument greatly increases power and may give deceptive results.

Dr. Hussey: There is chance of being deceived, but it is reduced to a minimum by the exactness of the instruments and their freedom from change. All the data we have on this subject come from Professor Jæger and Dr. Fincke. If such unvarying effects from potencies follow the taking of them, as proved by the instruments, we could not ask better proof. Adjourned,

C. P. JENNINGS, *Secretary.*

